# Novel Oversampling Technique for Improving Signal-to-Quantization Noise Ratio on Accelerometer-Based Smart Jerk Sensors in CNC Applications

**DOI:** 10.3390/s90503767

**Published:** 2009-05-19

**Authors:** Jose J. Rangel-Magdaleno, Rene J. Romero-Troncoso, Roque A. Osornio-Rios, Eduardo Cabal-Yepez

**Affiliations:** 1 Facultad de Ingeniería, Campus San Juan del Río, Universidad Autónoma de Querétaro / Río Moctezuma 249, Col. San Cayetano, 76807 San Juan del Río, Querétaro, Mexico; E-Mails: jjrangel@hspdigital.org (J.J.R.-M.); raosornio@hspdigital.org (R.A.O.-R.); 2 HSPdigital Research Group, División de Ingenierías, Campus Irapuato-Salamanca, Universidad de Guanajuato / Carr. Salamanca-Valle km 3.5+1.8, Comunidad de Palo Blanco, 36700 Salamanca, Guanajuato, Mexico; E-Mail: ecabal@hspdigital.org (E.C.-Y.)

**Keywords:** jerk, acceleration, smart sensors, SQNR, oversampling

## Abstract

Jerk monitoring, defined as the first derivative of acceleration, has become a major issue in computerized numeric controlled (CNC) machines. Several works highlight the necessity of measuring jerk in a reliable way for improving production processes. Nowadays, the computation of jerk is done by finite differences of the acceleration signal, computed at the Nyquist rate, which leads to low signal-to-quantization noise ratio (SQNR) during the estimation. The novelty of this work is the development of a smart sensor for jerk monitoring from a standard accelerometer, which has improved SQNR. The proposal is based on oversampling techniques that give a better estimation of jerk than that produced by a Nyquist-rate differentiator. Simulations and experimental results are presented to show the overall methodology performance.

## 1. Introduction

Nowadays, jerk monitoring has become a major issue in computerized numeric controlled (CNC) machines. Jerk is defined as the first derivative of acceleration and it provides information related to sudden changes in vibration levels of machinery. There are several works aimed to generate jerk limited trajectories and motion profiles for CNC machines and robotics. For instance, Osornio-Rios *et al.* [1] presented the implementation of higher degree polynomial acceleration profiles for peak jerk reduction in servomotors, Erkorkmaz and Altintas [2] developed a jerk limited trajectory generation and quintic spline interpolation for high-speed CNC machines and Dong *et al.* [3] showed a feed-rate optimization with jerk constraints for generating minimum-time trajectories for robotics. Moreover, there are a number of proposals focused on improving the CNC machining processes by monitoring system dynamics (involving position, speed, acceleration and jerk on servomotor actuated axes) and limiting vibration levels to increase tool life and reduce overall costs. The desired characteristics that next generation CNC machines should include are reviewed by Mekid *et al.* [4], and Lorenzer *et al.* [5] presented the modeling and evaluation of reconfigurable machines where jerk monitoring is of major relevance. These works highlight the necessity of measuring jerk (among other dynamic related variables) in a reliable way for improving production processes.

There are very few proposals for the development of a sensor that directly measures jerk like those reported by Nobuhiko *et al.* [6] and Fujiyoshi *et al.* [7], and there is also a lack of commercially available jerk sensors. In order to palliate this lack of jerk sensors two methodologies have been proposed: the sensorless and the accelerometer-based approaches. The sensorless approach reads the position information from an optical encoder, generally attached to all sevomotors in the axis control loop of the CNC machine, and then successively derivates the data to obtain an estimation of speed, acceleration, and jerk. This methodology has several disadvantages, as demonstrated by Chang and Chang [8], because derivatives are calculated using finite differences that corrupt information. By using wavelets, de Santiago-Perez *et al.* [9], and finite impulse response filters, Morales-Velazquez *et al.* [10], showed that it is possible to efficiently derivate the jerk signal from the optical encoder; nevertheless, the information provided by these researches is limited to the dynamics on the servomotor controlled axis and the methodology does not provide information on the induced vibrations due to the cutting process. On the other hand, accelerometer-based jerk monitoring takes into account the axis dynamics as well as the induced vibrations, but the derivation process to estimate the jerk from the acceleration leads to low signal-to-quantization noise ratio (SQNR) during the finite-difference computation of jerk [7,11].

Smart sensors that include in their functionalities signal processing, communication, and integration capabilities have become widely used in countless applications. The term “smart sensor” is employed according to the functionality classification given in Rivera *et al.* [12], from the definitions of the Institute of Electrical and Electronics Engineers [13-14]. A few examples, among many available, on the development of smart sensors are: Hernández [11], presented a survey on optimal signal processing techniques applied to improve the performance of mechanical sensors in automotive applications, focusing on the signal processing capabilities of smart sensors; Rivera *et al.* [12] developed a progressive polynomial algorithm for self-adjustment and optimal response in intelligent sensors, focusing their work on non-linear signal processing and present a microcontroller-based implementation; in another example described by Hernández [15], the response of several accelerometers in a car under performance tests is improved by using Kalman filtering, focusing the work in signal processing. These few samples highlight the relevance of smart-sensor development in recent times.

The novelty of this work is the development of a smart sensor for jerk monitoring from a standard accelerometer, which has improved SQNR. The proposal is based on oversampling techniques that uniformly distribute the quantization noise throughout the extended bandwidth, then filter the frequency-band where the signal information is contained, and further decimate (undersample) the data stream to give a better estimation of jerk than that produced by a Nyquist-rate discrete differentiator. Simulations and experimental results are presented to show the overall methodology performance.

## 2. Theoretical Framework

**Figure 1.**
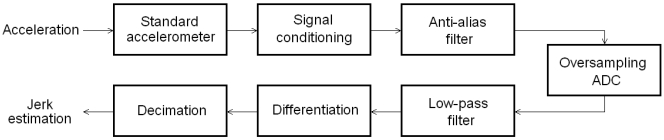
Block diagram of the smart sensor for jerk monitoring.

Figure 1 shows the block diagram of the proposed smart sensor for jerk monitoring. The system uses a standard accelerometer as primary sensor to measure acceleration. Signal conditioning and anti-alias filtering is then applied. Afterwards, the signal is converted to digital at an oversampling rate in the analog-to-digital converter (ADC). The quantized oversampled information is then filtered and differentiated to reduce the quantization noise. Finally, the resulting signal is decimated to give the estimation of jerk.

### 2.1. Derivative of a Quantized Signal

As it has been shown by several authors [7-11], the direct derivation of a quantized signal gives a poor estimation of the derivative. This is because the derivation is done by finite differentiation of the quantized signal, as stated in Equation (1), where the discrete-time derivative *j(k)* is the finite difference Δ *a(k)* taken between two consecutive quantized samples *a(k)* and *a(k-1)*:

(1) j(k)=Δa(k)=a(k)−a(k−1)

**Figure 2.**
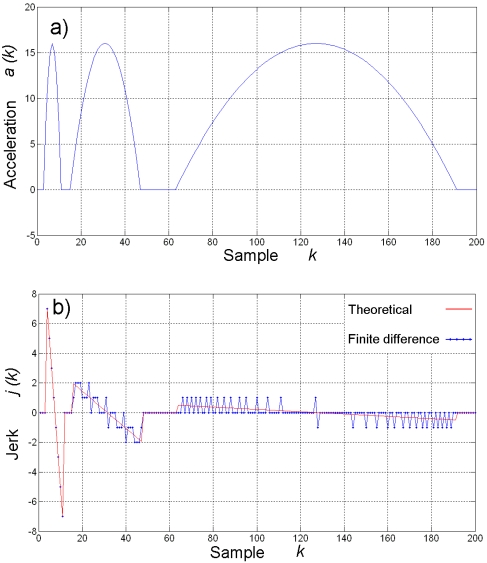
(a) Piecewise quadratic acceleration profile. (b) Theoretical and finite-difference derivatives.

Being *a(k)* an *n*-bit quantized signal that takes values in the range: [–2^n–1^, 2^n–1^–1], the resolution of its finite difference is inversely dependent on the change rate of *a(k)*. This means that the resolution of *j(k)* remains the same for a quick-changing *a(k)*, but the resolution is decreased for a slow-changing *a(k)*. To illustrate this effect consider Figure 2. Figure 2a shows a typical quadratic acceleration profile *a(k)* with quick-, medium-, and slow-changing piecewise segments. On the other hand, Figure 2b shows the theoretical and the finite-difference derivatives *j(k)*.

From Figure 2b, it can be seen that theoretical and finite-difference derivatives are essentially the same for a quick-changing acceleration profile. For a medium-changing acceleration profile, the finite-difference derivative resembles the theoretical derivative, but quantization noise starts to be significant. However, when the acceleration profile has a low-changing rate, the finite-difference derivative is highly corrupted with quantization noise, when compared against the theoretical derivative. Then, if finite difference is used as estimation for the derivative, it is necessary to improve the signal-to-quantization noise ratio of the process.

### 2.2. Signal-to-Quantization Noise Ratio

SQNR, in *dB*, for an *n*-bit quantized band-limited signal with bandwidth *BW* at a sampling rate *f_s_* is given by Equation (2):

(2) $${\text{SQNR}}_{dB}=20\log_{10}2^{n}\frac{f_{s}}{2BW}$$

**Figure 3.**
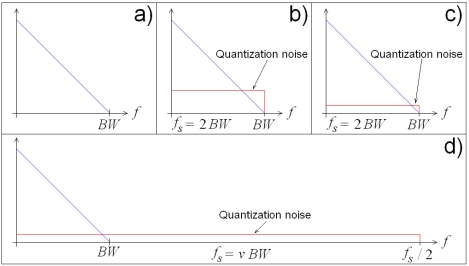
Signal spectra. (a) Noiseless. (b) *n*-bit resolution at Nyquist rate. (c) Increased resolution at Nyquist rate. (d) *v*-times oversampled with *n*-bit resolution.

This equation suggests that SQNR can be improved in two ways: by increasing resolution *n*, or by increasing sampling rate *f_s_* with further filtering. This improvement can be appreciated in Figure 3. Figure 3a shows the noiseless spectrum of a band-limited signal, Figure 3b shows the effect of the quantization noise over the *n*-bit resolution signal sampled at the Nyquist rate (absolute minimal sampling rate) *f_s_* = 2*BW*, Figure 3c shows the improvement when resolution is increased while maintaining the Nyquist sampling rate, and Figure 3d shows the improvement when maintaining resolution *n* with ν-times oversampling.

To increase the system resolution for improving SQNR is not always possible in certain applications because the cost could increase beyond the economical restrictions. On the other hand, for low-frequency applications such as jerk monitoring in CNC machines [9,10] where sampling rates are in the order of few *kHz*, the resolution can be maintained while the improvement is given by oversampling; this is possible considering that there are plenty of low-cost, commercially-available, sampling ADC circuits that easily handle sampling rates in the order of 100 *kHz*. In order to take advantage of the SQNR by oversampling, further digital signal processing is necessary, but this can be achieved with low-cost field programmable gate arrays (FPGA) [1].

### 2.3. SQNR Improvement by Oversampling

**Figure 4.**
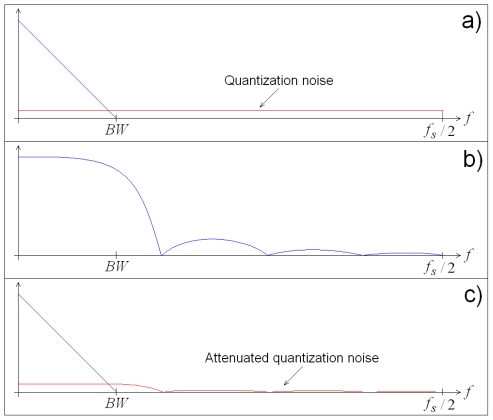
SQNR improvement with LPF. (a) Original oversampled signal. (b) Transfer characteristic of a real LPF. (c) Filtered signal.

To take advantage on the SQNR improvement by oversampling, it is necessary to low-pass filter (LPF) the signal in order to recover the significant information while suppressing the quantization noise that has been distributed along the oversampled frequency band. The transfer characteristic of the LPF determine the suppression level to the excess of quantized noise; and because the ideal filter is unrealizable in practice, certain amount of quantization noise remains, making SQNR lower than the stated value in Equation (2). Figure 4 shows the effect of real LPF acting on the oversampled signal. Figure 4a contains the original oversampled signal, Figure 4b presents the transfer characteristic of a real LPF, and Figure 4c shows the filtered signal.

Once the oversampled signal is filtered, the derivation by finite differences can be applied for further decimation to give the estimation of the derivative with improved SQNR.

## 3. Simulation Results

In order to test the efficiency of the developed methodology, the simulation of a case study is presented. This study consists in processing a typical acceleration profile to derivate jerk at different oversampling rates.

### 3.1. Signal Processing

**Figure 5.**
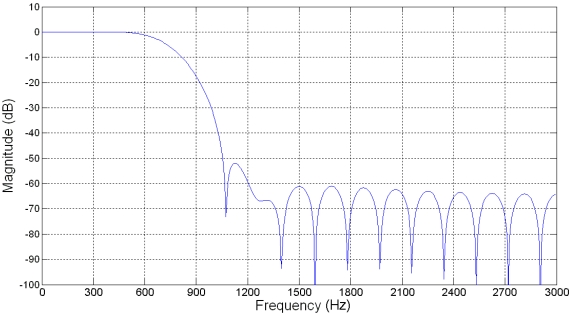
Frequency response of the FIR LPF at 4-times oversampling.

The digital processing of the signal consists of three stages: filtering, differentiation, and decimation. For this experiment, a 32^nd^ order, Hamming window, finite-impulse response (FIR) LPF [16] is proposed. The cut-off frequency is set at the original signal bandwidth *BW*, giving this filter 6 *dB* of attenuation at the cut-off frequency. Other filtering schemes can be utilized at this stage, having in mind the rejection characteristics on the oversampled band. Figure 5 shows the frequency response of the FIR LPF magnitude, for the 4-times oversampling case at a sampling frequency *f*_s_ = 6,000 *Hz*. This filter has an excess of 50 *dB* attenuation at the suppression band.

Once the oversampled signal is filtered, the next step is to obtain the derivative by finite differences as stated in Equation (3), being *a(k)* the oversampled acceleration signal, *j(k)* the estimated jerk, and *v* the oversampling rate:

(3) j(k)=a(k)−a(k−v)

The decimation is done by directly undersampling the estimation from the finite difference stage, giving one sample at the output for each set of ν consecutive samples at the input and discarding the others [16].

### 3.2. Study Case

**Figure 6.**
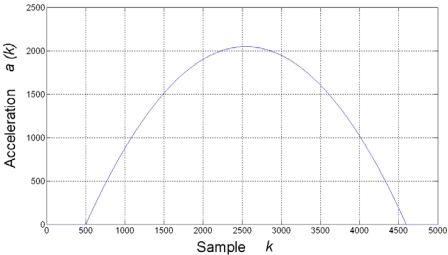
Typical slow-changing quadratic acceleration profile.

As it was demonstrated in Section 2.1, the finite-difference effects for estimating the derivative of a signal are more severe when the signal has a slow-changing rate; therefore, a slow-changing quadratic acceleration profile is utilized for testing the proposed methodology, as shown in Figure 6. This profile was generated with a positive-only quadratic waveform at 12-bit resolution and spread along 4,096 samples. Finite differences of this profile has an expected absolute quantized maximum of 2, when these differences are calculated directly, giving an effective resolution of around 2 bits for the estimation, which is highly corrupted with quantization noise. The methodology is applied to demonstrate its efficiency by improving the effective resolution of the estimation.

**Figure 7.**
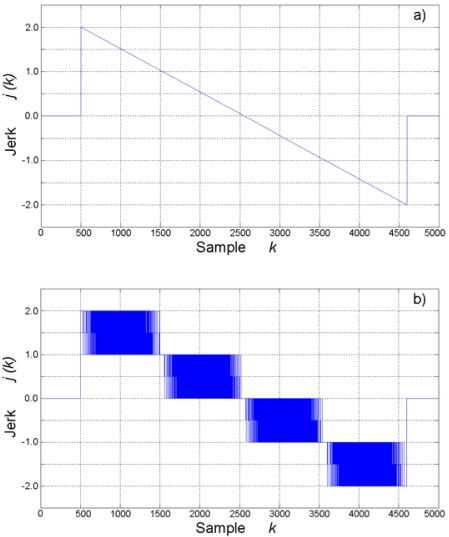

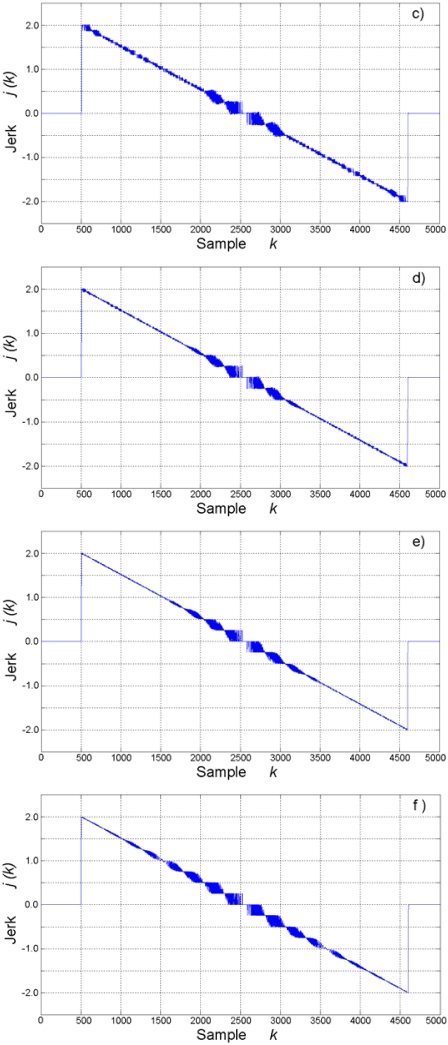
Jerk estimation. (a) Theoretical. (b) Nyquist rate. (c) 4-times oversampling. (d) 8-times oversampling. (e) 16-times oversampling. (f) 32-times oversampling.

Figure 7 shows the obtained results for jerk estimation from the acceleration profile. The theoretical jerk is shown in Figure 7a. Figure 7b shows the jerk as estimated by finite differentiation at the Nyquist sampling rate. Figures 7c-f contains the estimated jerk with the proposed methodology for 4-, 8-, 16-, and 32-times oversampling, respectively. As expected, direct finite differentiation for jerk estimation gives a highly corrupted signal that takes quantized integer values in the range: [-2, 2]. Jerk estimation with the proposed methodology greatly improves resolution on results, even for the 4-times oversampling rate.

**Figure 8.**
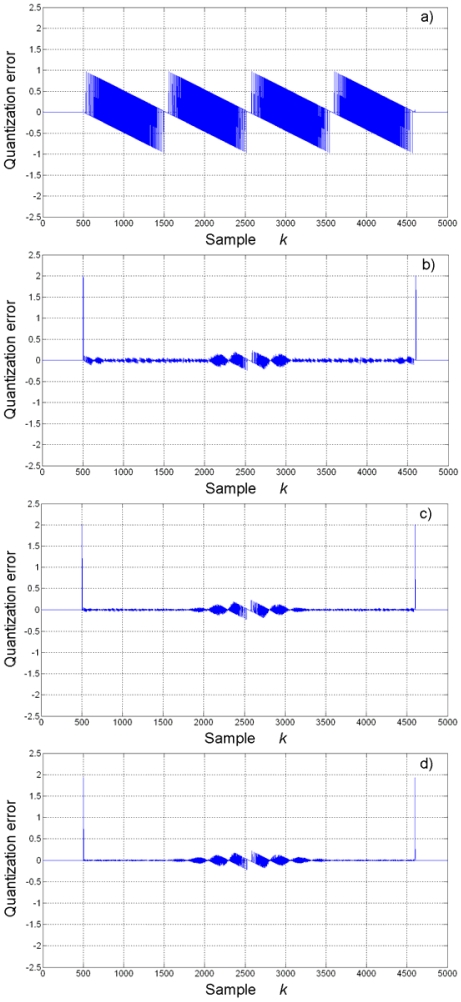

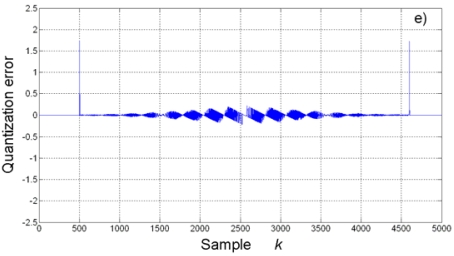
Quantization error of jerk estimation. (a) Nyquist rate. (b) 4-times oversampling. (c) 8-times oversampling. (d) 16-times oversampling. (e) 32-times oversampling.

The quantization error, calculated by subtracting the estimated jerk from the theoretical waveform, is shown in Figure 8. The quantization noise in the jerk estimation at Nyquist sampling rate (Figure 8a) is noticeable. Quantization noise presents significant reduction with the proposed methodology from 4-times oversampling and up. Quantization noise is further reduced at higher oversampling rates, up to a certain level, where the oversampling no longer improves the SQNR. For instance, SQNR is improved from 4-times up to 16-times oversampling, but the improvement is no longer evident at 32-times oversampling.

**Figure 9.**
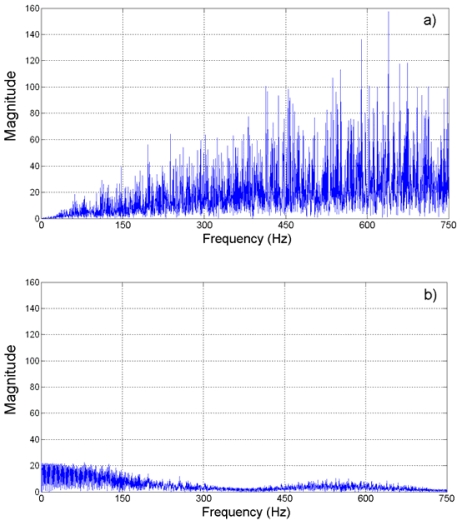

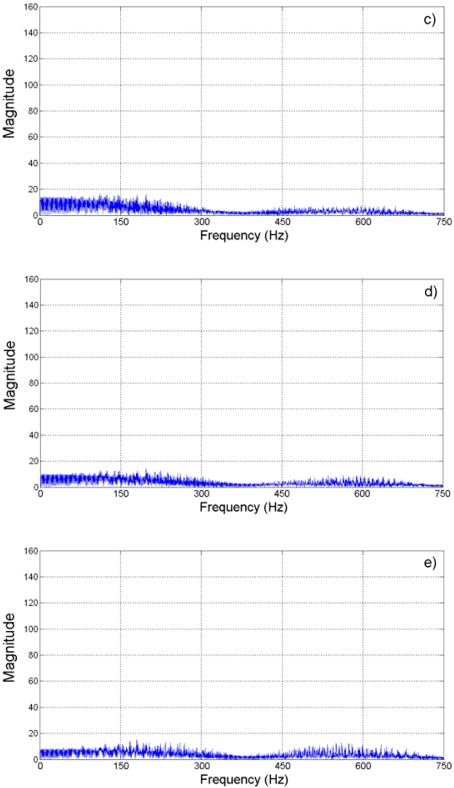
Spectra of quantization error for jerk estimation. (a) Nyquist rate. (b) 4-times oversampling. (c) 8-times oversampling. (d) 16-times oversampling. (e) 32-times oversampling.

On the other hand, the spectra of these quantization errors are shown in Figure 9. From this figure, it can be said that the spectral contents of quantization error at the Nyquist sampling rate is higher that the spectral contents with the oversampling approach. Then again, this improvement is present up to a certain level. Table 1 summarizes the SQNR improvement, in *dB*, for different oversampling rates in this case of study.

## 4. Experimental Results

This section presents the application of the proposed methodology to estimate jerk in a single axis of a CNC machine. The axis dynamics are controlled by a digital controller such as [1], to give a known acceleration profile and then experimental results are compared against the theoretical profile.

### 4.1. Instrumentation System

**Figure 10.**
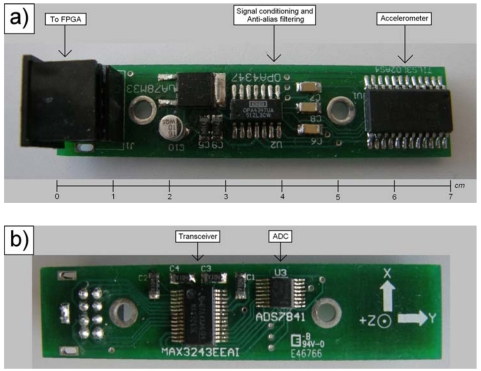
Instrumentation system PCB. (a) Top view. (b) Bottom view.

The proposed methodology for jerk estimation can be applied to any accelerometer. In our case a 3-axis LIS3L02AS4 accelerometer from STMicroelectronics [17] was used. The accelerometer has a user-selectable full scale of ± 2*g*/ ± 6*g* (*g* = 9.81 m/s^2^); a 5×10^-4^*g* resolution over a 100 *Hz* bandwidth; and a bandwidth of 1.5 *kHz* for all axes. The accelerometer is mounted in a PCB with the signal conditioning and anti-alias filtering, as recommended by the manufacturer. This PCB also contains a 4-channel, 12-bit sampling ADC from Texas Instruments ADS7841 [18], with a 200 *kHz* maximum sampling rate for each channel. The communication between the instrumentation system and the FPGA signal processing unit is done with a MAX3243 transceiver. Figure 10 shows the top and bottom views of the instrumentation system PCB.

The operating parameters of the instrumentation system for the experiment are set as follows: acceleration range of ± 2*g* (± 19.62 m/s^2^); 12-bit resolution at the ADC; 0.66 V/*g* sensitivity; one acceleration axis monitoring; anti-alias filter tuned to give a signal bandwidth *BW* = 750 *Hz*; Nyquist sampling rate *f_s_* = 1500 *Hz*; and 4-, 8-, 16-, and 32-times oversampling rates.

### 4.2. CNC Machine

**Figure 11.**
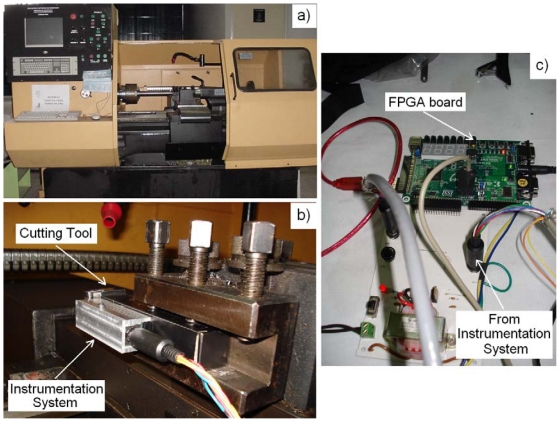
Experiment setup. (a) Retrofitted CNC lathe. (b) Instrumentation system mounting. (c) FPGA-based signal processing unit.

The instrumentation system with the accelerometer is encased in aluminum and mounted near the cutting tool of a retrofitted to CNC lathe. It is recommended to locate the accelerometer as close as possible to the cutting tool to properly sense chatter during the cutting process [19]. Figure 11a shows the retrofitted CNC lathe, Figure 11b shows the encased instrumentation system, mounted near the cutting tool, and Figure 11c shows the FPGA-based signal processing unit.

### 4.3. Signal Processing Unit

**Figure 12.**
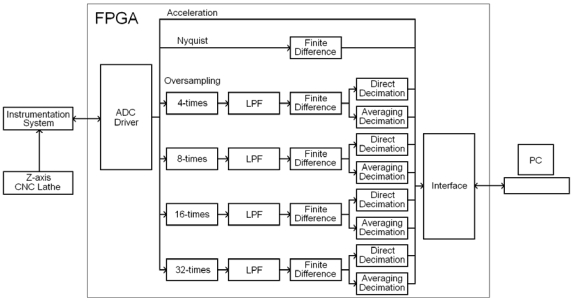
Block diagram of the FPGA-based signal processing unit.

The signal processing unit is implemented into a 200,000-gate Xilinx Spartan-3 FPGA and the block diagram of the implementation can be found in Figure 12. The implementation has six stages: ADC driver, oversampling, LPF, finite difference, decimation, and interface. The ADC driver stage handles the data acquisition and conversion at the 32-times oversampling rate and passes the information to the following stage. The oversampling stage takes the incoming acceleration data at the following rates: Nyquist, 4-, 8-, 16-, and 32-times oversampling. The 32^nd^ order FIR LPF, as defined in Section 3.1, are implemented in the filtering stage. The finite-difference stage computes the derivative estimation according to Equation (3). The decimation stage decimates the oversampled signals with two algorithms: direct decimation, and averaging decimation. The direct decimation algorithm takes a single datum from each incoming set of ν data points and discards the others. The averaging decimation algorithm computes the average of ν consecutive data points and gives a single result for every ν points. The interface stage sends results from acceleration, Nyquist-rate jerk estimation and oversampling rate estimations to a PC for storage and further analysis.

### 4.4. Acceleration Profile

**Figure 13.**
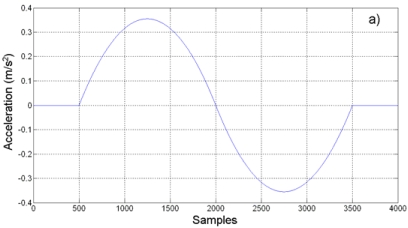

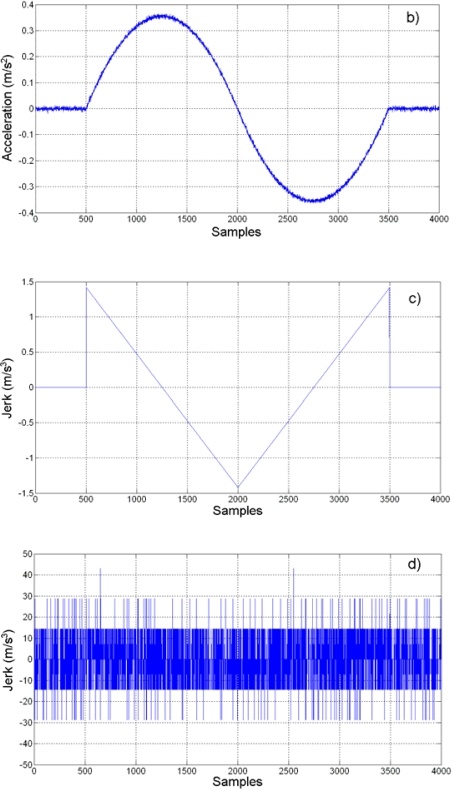
Profiles (a) Theoretical biquadratic acceleration profile. (b) Measured acceleration. (c) Theoretical jerk profile. (d) Jerk estimation at Nyquist sampling rate by finite differences.

The acceleration/deceleration profile for experimentation is a biquadratic profile to produce a displacement of 0.5 *m* at the lathe *Z*-axis in a period of 2*s*, representing a typical movement for axis positioning in CNC machines. This profile produces an absolute peak acceleration of 0.355 m/s^2^. At a Nyquist sampling rate of *f_s_* = 1,500 *Hz*, the profile contains 3,000 samples for the movement. Figure 13a shows the theoretical biquadratic acceleration profile. The measured acceleration at the Nyquist rate is shown in Figure 13b where it can be seen that it is slightly embedded with noise from the cutting process. The theoretical jerk can be found in Figure 13c, whereas the estimated jerk by finite differences at the Nyquist rate is shown in Figure 13d. From Figures 13c and 13d it is easily seen that the Nyquist-rate jerk estimation is highly corrupted, as expected.

### 4.5. Results

**Figure 14.**
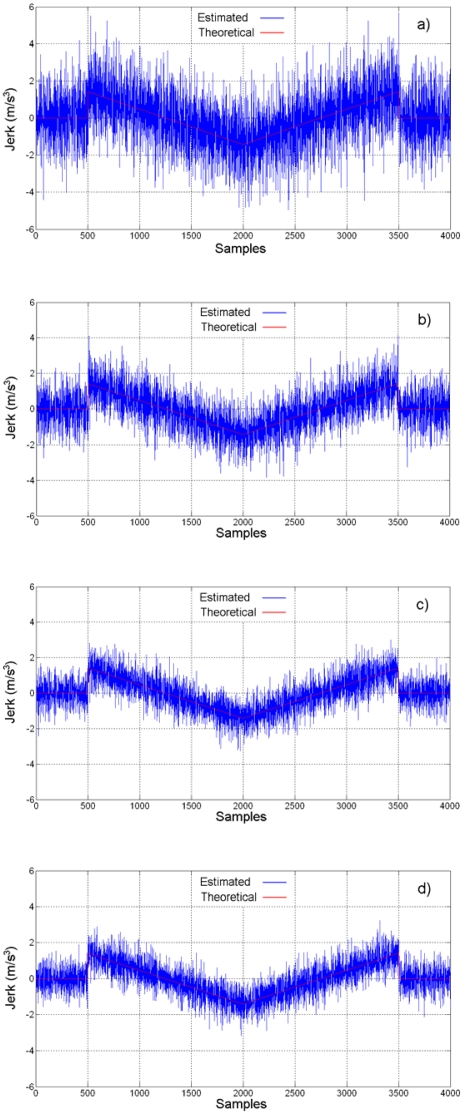
Jerk estimation with oversampling and direct decimation. (a) 4-times. (b) 8-times. (c) 16-times. (d) 32-times.

**Figure 15.**
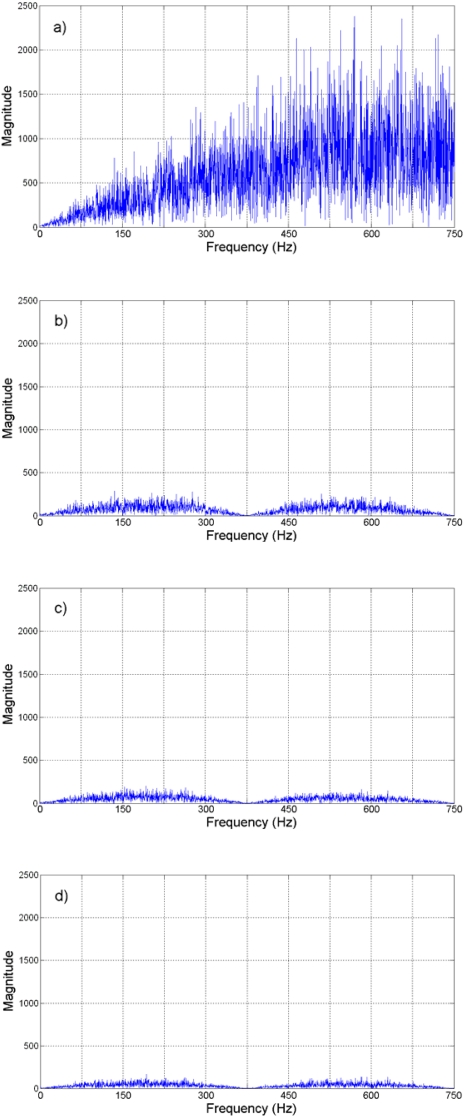

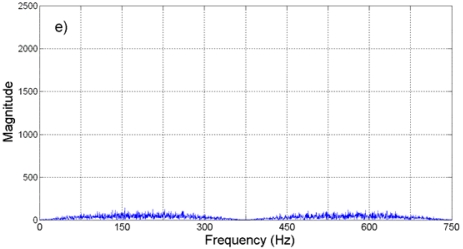
Direct decimation jerk error spectra. (a) Nyquist rate. (b) 4-times oversampling. (c) 8-times oversampling. (d) 16-times oversampling. (e) 32-times oversampling.

**Figure 16.**
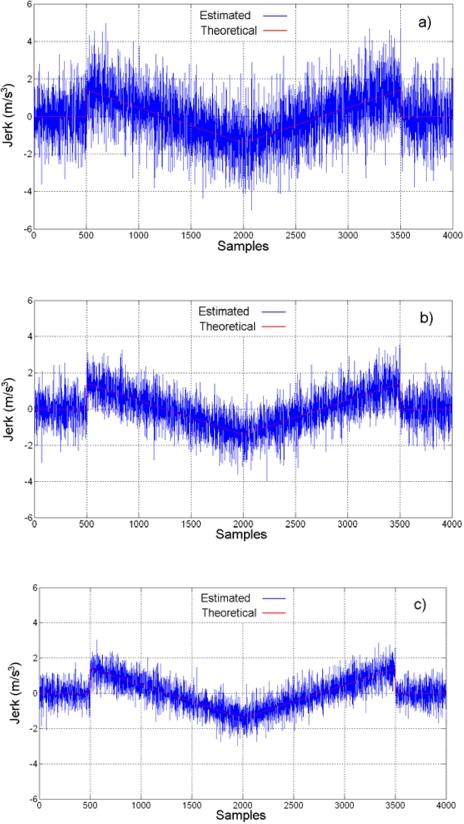

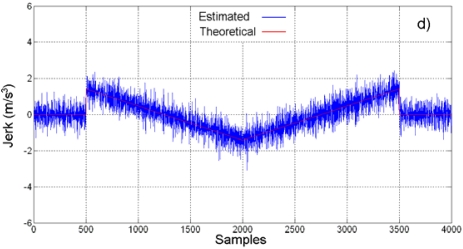
Jerk estimation with oversampling and averaging decimation. (a) 4-times. (b) 8-times. (c) 16-times. (d) 32-times.

**Figure 17.**
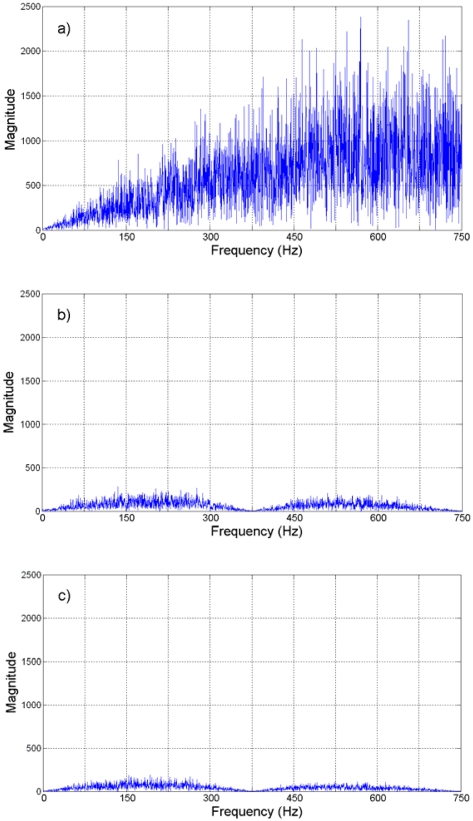

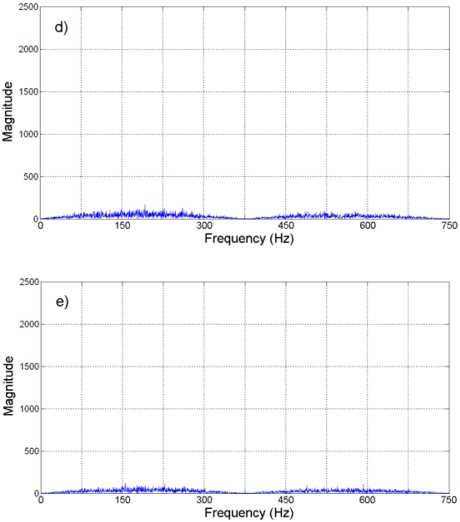
Averaging decimation jerk error spectra. (a) Nyquist rate. (b) 4-times oversampling. (c) 8-times oversampling. (d) 16-times oversampling. (e) 32-times oversampling.

Jerk estimation with the oversampling methodology for the experiment is shown in Figure 14 at different oversampling rates, using the direct decimation algorithm. The corresponding error spectra of these results, along with the spectrum of the Nyquist-rate estimation, are shown in Figure 15. On the other hand, Figure 16 contains the jerk estimation at different oversampling rates with the averaging decimation algorithm, and their corresponding error spectra are shown in Figure 17.

Table 2 summarizes the SQNR improvement, in *dB*, for different oversampling rates in this experiment.

### 4.6. Discussion

Results in Figures 14 to 17 show that the oversampling methodology for derivative estimation gives better results than finite differences at Nyquist rate. The estimated jerk profile with oversampling greatly resembles the theoretical jerk profile. The presence of noise is unavoidable at real measurements and it affects results; yet, it is possible to obtain a good estimation of the jerk profile by oversampling, whereas it is not possible with the Nyquist-rate finite differences (Figure 13d compared with Figures 14 and 16). The decimation algorithm also plays a role in the quality of the estimation as is deduced from the SQNR data in Table 2, where decimation by averaging is slightly better than the direct decimation; this can be appreciated when comparing Figures 14 with 16 and Figures 15 with 17. The developed technique focuses on the SQNR improvement. The noise on the resulting jerk estimation in Figures 14 and 16 is due to the cutting process. The reduction of the noise due to the cutting process is beyond the scope of this research; yet, noise-reduction techniques such as [9, 10] can be utilized to further improve the jerk estimation.

## 5. Conclusions

The development of a novel smart sensor for jerk estimation from acceleration with an oversampling technique for improving SQNR in CNC applications is presented. The primary sensor is a standard accelerometer and the jerk estimation is done through oversampling signal processing techniques. SQNR improvement is achieved by oversampling the acceleration signal, then successively applying low-pass filtering, finite differences, and decimation. Simulations and experimentation is done to test the methodology efficiency. Simulations show that SQNR improvement is over 23 *dB* when applying the proposed methodology and compared against the Nyquist-rate finite differences. Experimentation over a typical acceleration/deceleration profile in CNC machines shows that the standard Nyquist-rate finite differences, as reported by literature [6-10], for estimating jerk from acceleration are not suitable for this purpose because the resulting jerk profile is highly corrupted by quantization and measuring noise. On the other hand, the developed technique shows its efficiency in producing a recognizable jerk estimation that greatly resembles with theoretical jerk, under real CNC machine operation.

Another contribution of this work is the FPGA-based implementation of the signal processing unit in hardware for low-cost and real-time processing at the smart jerk sensor. This implementation also shows that FPGA are a suitable solution for embedded signal processing in developing smart sensors. The developed technique can be applied for derivation of jerk from a standard accelerometer, in an efficient way, and also to estimate the derivative from other kinds of sensors. Further research can be done to reduce measuring noise in jerk estimation and also to mount a low-power FPGA on the smart sensor PCB for on-board hardware signal processing.

## Figures and Tables

**Table 1.** SQNR improvement at different oversampling rates.

**Oversampling rate (*v*)**	4	8	16	32
**SQNR improvement (***dB*)	23.12	27.47	30.22	30.01

**Table 2.** SQNR improvement at different oversampling rates.

**Oversampling rate (*v*)**	**4**	**8**	**16**	**32**
**SQNR (*dB*) Direct decimation**	33.83	44.09	49.26	50.53
**SQNR (*dB*) Averaging decimation**	38.71	45.89	51.90	55.91
